# Phase angle was associated with improved cognitive function over time with mediation from pulse wave velocity in type 2 diabetes mellitus

**DOI:** 10.1002/alz70855_098722

**Published:** 2025-12-23

**Authors:** Serena Low, Kiat Sern Goh, Angela Moh, Keven Ang, Chee Fang Sum, Tavintharan Subramaniam, Su Chi Lim

**Affiliations:** ^1^ Khoo Teck Puat Hospital (Alexandra Health Pte Ltd), Singapore, Singapore, Singapore; ^2^ Changi General Hospital, Singapore, Singapore, Singapore; ^3^ Admiralty Medical Centre/ Khoo Teck Puat Hospital, Singapore, Singapore, Singapore

## Abstract

**Background:**

Phase angle (PhA) is the angle of vector which comprises the body's resistance and reactance. It reflects nutritional status with higher levels signifying healthier cell membrane and higher muscle mass. Although higher PhA was associated with better clinical outcomes, the association between PhA and cognition as well as the underlying pathophysiological mechanism are unknown. Arterial stiffness confers higher risk of cognitive impairment due to disruption of cerebral blood flow, particularly in patients with type 2 diabetes(T2D). We investigated the association between PhA and cognitive function with lower arterial stiffness as a potential mediator in T2D.

**Method:**

We conducted a prospective cohort study of 908 patients from SMART2D cohort. Whole body PhA at baseline was measured by bioelectrical impedance analysis. We assessed cognitive function with Repeatable Battery for Assessment of Neuropsychological Status (RBANS). Carotid‐femoral pulse wave velocity (PWV), a gold‐standard measure of arterial stiffness, was measured with applanation tonometry. Linear regression and linear mixed model were used to examine the cross‐sectional and longitudinal associations between PhA and cognitive function, adjusting for demographics, clinical covariates, presence of APOE_Ɛ_4 allele and medications.

**Result:**

The participants’ mean age was 60.6 ± 7.8 years. Higher PhA was cross‐sectionally associated with higher RBANS total score with adjusted coefficient 1.17 (95%CI 0.40 to 1.94; *p* = 0.003). 834 patients were followed up to 8.4 years. Higher PhA was longitudinally associated with higher RBANS total score with adjusted coefficient 1.18 (95%CI 0.44 to 1.93; *p* = 0.002). There was also positive association between PhA and domain scores in visuospatial‐construction (coefficient 1.40; 95%CI 0.31 to 2.49; *p* = 0.012), attention (coefficient 1.83; 95%CI 0.80 to 2.86; *p* <0.001) and delayed memory (coefficient 1.86; 95%CI 0.60 to 3.12; *p* = 0.004) in adjusted longitudinal analyses. Mediation analysis revealed that PWV accounted for 10% of the association between PhA and follow‐up RBANS total score (*p* = 0.044)

**Conclusion:**

Our findings revealed previously unobserved findings of longitudinal associations between PhA with cognitive function globally with PWV as a potential mediator. The associations were also evident in multiple cognitive domains, including memory, attention and visuospatial‐construction. PhA may be a potential intervention target for early prevention of cognitive impairment in T2D in future clinical practice.

